# Robotic Laser Tissue Soldering for Atraumatic Soft Tissue Fusion Guided by Fluorescent Nanothermometry

**DOI:** 10.1002/advs.202406671

**Published:** 2024-11-21

**Authors:** Oscar Cipolato, Tobias Leuthold, Marius Zäch, Georg Männel, Sam Aegerter, Calinda Sciascia, Alexander Jessernig, Marco von Salis, Sima Sarcevic, Jachym Rosendorf, Vaclav Liska, Dennis Kundrat, Romain Quidant, Inge K. Herrmann

**Affiliations:** ^1^ Nanoparticle Systems Engineering Laboratory Institute of Energy and Process Engineering (IEPE) Department of Mechanical and Process Engineering (D‐MAVT) ETH Zurich Sonneggstrasse 3 Zurich 8092 Switzerland; ^2^ Particles Biology Interactions Laboratory Department of Materials Meet Life Swiss Federal Laboratories for Materials Science and Technology (Empa) Lerchenfeldstrasse 5 St. Gallen 9014 Switzerland; ^3^ The Ingenuity Lab University Hospital Balgrist Forchstrasse 340 Zurich 8008 Switzerland; ^4^ Faculty of Medicine University of Zurich Rämistrasse 71 Zurich 8006 Switzerland; ^5^ Fraunhofer Research Institution for Individualized and Cell‐Based Medical Engineering IMTE Mönkhoferweg 23562 Lübeck Germany; ^6^ Department of Surgery Faculty of Medicine in Pilsen Charles University Alej Svobody 923/80 Pilsen 32300 Czech Republic; ^7^ Biomedical Center Faculty of Medicine in Pilsen Charles University Alej Svobody 1655/76 Pilsen 32300 Czech Republic; ^8^ Nanophotonic Systems Laboratory Institute of Energy and Process Engineering (IEPE) Department of Mechanical and Process Engineering (D‐MAVT) ETH Zurich Sonneggstrasse 3 Zurich 8092 Switzerland

**Keywords:** feedback control, laparoscopy, minimally invasive surgery, nanomaterials, suturing

## Abstract

Minimally invasive surgical techniques, including endoscopic and robotic procedures, continue to revolutionize patient care, for their ability to minimize surgical trauma, thus promoting faster recovery and reduced hospital stays. Yet, the suturing of soft tissues ensuring damage‐free tissue bonding during these procedures remains challenging due to missing haptics and the fulcrum effect. Laser tissue soldering has potential in overcoming these issues, offering atraumatic seamless tissue fusion. To ensure the precision and safety of laser tissue soldering, the study introduces feedback‐controlled fluorescent nanothermometry‐guided laser tissue soldering using nanoparticle‐protein solders within endoscopic and robotic contexts. Temperature‐sensitive fluorescent nanoparticles embedded in the solder provide surgeons with immediate feedback on tissue temperatures during laser application, all while within the confines of minimally invasive (robotic) surgical setups. By integrating fluorescent nanothermometry‐guided laser tissue surgery into endoscopic and robotic surgery, the study paves the way for a new approach for safe and atraumatic soft tissue joining, especially in regions where traditional suturing is unfeasible.

## Graphical abstract

Minimally invasive techniques like endoscopic and robotic surgery reduce trauma and speed recovery, yet soft tissue suturing remains challenging. Laser tissue soldering offers atraumatic, suture‐free bonding, using fluorescent nanothermometry‐enhanced protein solders for real‐time temperature feedback, ensuring precision and safety. This method improves soft tissue fusion in minimally invasive settings, particularly in areas where traditional suturing is impractical.

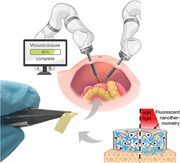

## 1 Introduction

Minimally invasive surgery (MIS), i.e., surgical procedures that use various techniques to minimize the size of the needed incisions, has been growing in popularity for many years, with case increases of up to 462% between 2000 and 2018.^[^ ^1^ ^]^ However, due to the limited working space inside the patient associated with this surgical technique, the disadvantages of suturing become more pronounced.^[^ ^2^ ^]^ Difficulties in suturing in minimally invasive surgery are greatly amplified by the fulcrum effect (the result of pivoting instruments at varying depths), minimal or even absence of haptic feedback, amplified tremor, and a reduced surgical field view, among other factors.^[^ ^3^, ^4^ ^]^ To overcome some of the limitations in conventional MIS, robotic MIS has been introduced to the operating rooms with great success in the last decades.^[^ ^5^, ^6^ ^]^ However, sutures and staples have their own set of intrinsic drawbacks, particularly in soft tissue injuries to the liver, the intestine, blood vessels, nerves, or the dura mater. Suturing and stapling techniques are not only susceptible to inflammation, infection, and slow healing, but they can also cause tissue trauma^[^ ^7^ ^]^ and lead to dangerous fluid leaks.^[^ ^8^, ^9^, ^10^ ^]^

To overcome the current limitations of suturing in endoscopic and robotic surgery, various surgical glues and wet tissue adhesives have been investigated.^[^ ^11^, ^12^ ^]^ While potentially promising, limitations^[^ ^13^, ^14^ ^]^ include toxicity (e.g., cyanoacrylates),^[^ ^15^ ^]^ immunogenicity, low adhesion (e.g., fibrin‐ or poly(ethylene glycol)‐based glues),^[^ ^16^ ^]^ and swelling (e.g., hydrogel‐based sealants).^[^ ^11^, ^12^, ^17^ ^]^ Most of these technologies have found their main application in open surgery and they are poorly applicable for minimally invasive procedures. The physical dimensions and application techniques of these adhesives frequently pose challenges for seamless adaptation to a minimally invasive environment.

Laser tissue soldering represents a promising alternative: it is a surgical technique that combines laser light with a solder paste, a temperature‐activatable adhesive material, usually protein‐based, to create strong watertight bonds.^[^ ^18^, ^19^, ^20^ ^]^ The attractiveness of soldering lies in its ability to rapidly form bonds with good strength,^[^ ^21^, ^22^, ^23^ ^]^ low inflammatory response,^[^ ^24^ ^]^ reduced scar tissue formation,^[^ ^25^ ^]^ and reduced access to pathogens thanks to the creation of a waterproof seal.^[^ ^26^ ^]^ Furthermore, this technique is, in principle, readily adaptable for minimally invasive procedures.^[^ ^10^ ^]^ The minimal amount of material required for bonding, the straightforward guiding of laser light to the bonding location, and the simplicity of the technique — which does not require intricate motions or exhaustive training — all contribute to its feasibility.^[^ ^27^, ^28^ ^]^ Although laser tissue soldering holds great promise, it remains experimental.^[^ ^19^ ^]^ The technique's success hinges crucially on the precise temperature management during the procedure. For optimal bonding, temperatures between 60 and 80 °C are required, yet the surrounding tissue must remain significantly cooler to avoid irreversible tissue damage. Insufficient temperatures during soldering result in inadequate protein‐tissue bonding, while excessive heat can damage surrounding tissues and delay healing.^[^ ^18^ ^]^ Thus, mastering the confinement and control of the temperature is critical for clinical translation.

In this work, we introduce a new minimally invasive wound closure technique for (robotic) MIS, based on controlled laser tissue soldering guided by fluorescent nanothermometers able to create seamless water‐tight sealing. Nanothermometers have recently been successfully integrated into proteinaceous solders,^[^ ^29^ ^]^ and offer non‐invasive temperature measurements. The proteinaceous solder material can further be supplemented with nanoabsorbers, including gold nanorods or titanium nitride nanoparticles^[^ ^29^ ^]^ for optimal differential heating. As a critical step toward more accurate and less invasive soldering, we demonstrate automatic temperature control and smart laser power modulation for high‐performance laser tissue soldering in (robotic) MIS, enabled by an intelligent solder paste that contains NIR‐fluorescent nanothermometers and nanoabsorbers. Moreover, we present integrating computer‐vision based solder recognition along with multiple approaches to guide the surgery by measuring the nanothermometry‐based temperature distribution during soldering for optimal outcome. Finally, we demonstrate the crucial advantages of minimally invasive soldering in clinically‐relevant scenarios.

## 2 Results

### 2.1 Design of a Controlled Soldering Process for Minimally Invasive Procedures

**Figure 1:**
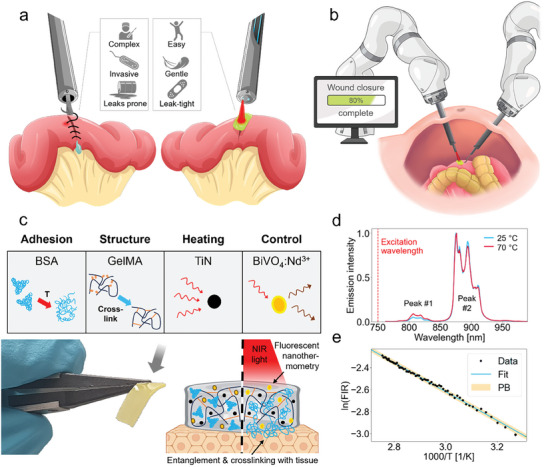
Controlled tissue soldering for seamless tissue fusion in MIS. a) Illustration showing the advantages of laser tissue soldering compared to conventional suturing of soft tissues. b) Schematic of laser tissue solder integration into robotic minimally invasive surgery. c) The components of the solder paste (shown in the photo) are illustrated schematically, along with a schematic of the soldering mechanism. d) Fluorescence spectra collected at two different nanothermometer temperatures (normalized intensity). The ratio of the two fluorescence emission peaks of BiVO_4_:Nd^3+^ are used for non‐contact temperature measurements. e) Calibration plot used to calculate the temperature from the fluorescence intensity ratio FIR (*a* = 1.33 ± 0.03, *b* = −1.32 ± 0.01 K^−1^ are the values of the fit *y*  = *a* + *b* × *x* with *y*  =  ln(*FIR*) and *x*  =  1/*T*; PB = Prediction Band).

To address the current limitations of soft tissue joining in MIS, the ideal approach should not only facilitate wound closure in a minimally invasive manner but also establish robust and reliable tissue bonds, all while preserving the integrity of the surrounding healthy tissue. Good reliability can be obtained only if the technique is easy to perform and relies on a robust environment‐independent mechanism. Moreover, it should be compatible with existing surgical tools of widespread use (**Figure** **1**a,b). Amongst the potential technologies for soft tissue repair in the constrained environment of MIS, laser tissue soldering offers a particularly promising approach. Especially using proteinaceous solders with integrated nanoabsorbers for optimal heat confinement, and nanothermometers for accurate temperature control during soldering, offers a route to seamless tissue fusion.

We designed an albumin‐based solder paste optimized for MIS, by using gelatin‐methacrylate (GelMA) as a structure‐giving moiety for optimal endoscopic and robotic application and manipulation of the paste. Addition of GelMA as a structure‐giving element is essential to avoid uncontrolled liquefaction of the solder material during manipulation. Additionally, TiN (for confined temperature increase) and BiVO_4_:Nd^3+^ nanoparticles (for nanothermometry) were integrated into the paste for optimal performance and integration into feedback controlled laser tissue soldering (Figure 1c). TiN nanoparticles are used as nanoheaters due to their broad absorption spectrum, high photothermal efficiency, stability, low cost, and biocompatibility.^[^ ^30^ ^]^ Flame‐made bismuth vanadate doped with neodymium (BiVO_4_:Nd^3+^) serve as fluorescent nanothermometers. Their merit lies in their excitation and emission wavelengths situated within the near‐infrared (NIR) biological window, ensuring safety and robust performance regardless of the environment (Figure 1d). The fluorescence intensity ratio (FIR) can be calculated from two BiVO_4_:Nd^3+^ emission peaks and correlated with the temperature through the Boltzmann thermal equilibrium equation (Figure 1e and Materials and Methods). Using a ratiometric approach brings clear advantages, as it makes the measurement unaffected by nanothermometer concentration, laser power, and weakly‐absorbing materials in the NIR situated between or on the paste and the measuring fiber, such as thin tissues or various bodily fluids. This is an important distinction from widely used thermometry methods that rely on intensity measurements in the Mid‐IR range (3–20 µm, and most commonly 3–5 µm or 8–14 µm),^[^ ^10^ ^]^ which are strongly affected by environmental factors. The absorption coefficient of water in this range is in the order of 10^4^–10^6^ m^−1^,^[^ ^31^ ^]^ ≈4 orders of magnitude higher than in the NIR range (absorption coefficient of water between 0.7 and 1.4 µm is in the order of 10^0^–10^2^ m^−1^). This means that with infrared thermometry a water layer of 1–100 µm on the paste or on the tip, something that readily occurs during MIS due to tissue manipulation, would mask the actual temperature, leading to incorrect measurements for conventional thermometry, while fluorescence nanothermometry measurements remain unaffected. It is noteworthy that the yellow‐green color of the paste arises from nanoparticle incorporation, not as a design feature. While this color improves visibility, it does not convey temperature information and can be less discernible amidst body fluids and internal structures. Therefore, fluorescence remains critical for both accurate detection and temperature control during the soldering process.

Soldering using the nanomaterial‐enhanced solder paste can readily be integrated into a (robotic or endoscopic) MIS setup. By integrating two optical fibers in an endoscopic instrument, the solder paste can be irradiated through one fiber and the fluorescence of the nanothermometers can be measured simultaneously by the second fiber, providing crucial information about the temperature during soldering in a contact‐free, minimally invasive manner (Figure 1b). Filtering of the light source and of the signal can be performed at the distal end of the optical fibers, away from the soldering location, allowing for increased flexibility on how to filter and process the signal collected from the surgical site within the patient. For example, the temperature information can be used to modulate the laser power to provide controlled and safe soldering, either in an automatized or user‐controlled fashion. Moreover, the optical fibers can be removed and replaced or sterilized, a crucial requirement for in vivo studies and use in clinical operating rooms.

### 2.2 Automatized Feedback for Controlled Heating

**Figure 2:**
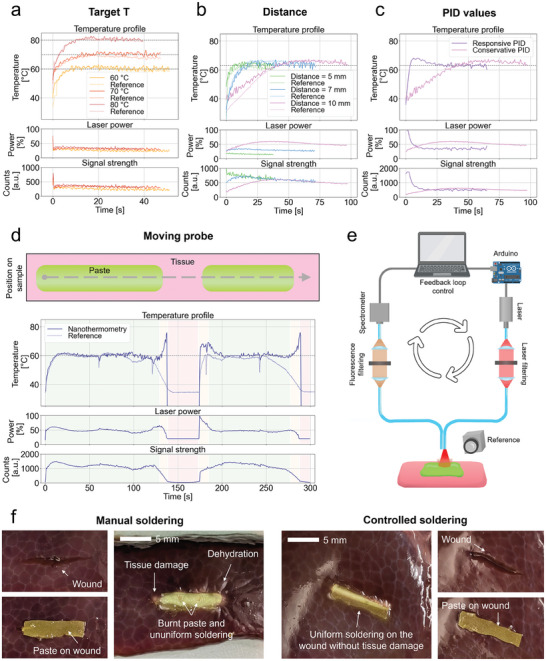
Automatized feedback for controlled local heating. a) Feedback‐controlled soldering allows for soldering at various temperatures (60, 70, 80 °C), achieving the target temperature in less than 10 s for temperatures needed in tissue soldering (top plot). Temperature control is kept by modulating the laser power (middle plot) and based on the signal strength (bottom plot). (*d* = 10 mm) b) The target temperature (here 63 °C) is reached also at different distances of the fibers from the solder paste, however, with a slower response at higher distances. c) The PID parameters can be tuned to obtain a responsive behavior, at the cost of an initial small temperature overshoot. (*d* = 10 mm, *T* = 63 °C) d) The feedback mechanism can be employed also while the optical fiber‐endoscope is moved over the wound (as illustrated in the top part): two pieces of solder paste are placed on a piece of pig intestine and the fibers are moved over them. The controller is also able to discriminate when the paste is being irradiated (green areas) and when it is above healthy tissue (red areas), automatically lowering the laser power to a safe level (15%). (*d* = 10 mm, *T* = 60 °C). e) Illustration of the feedback loop setup used for paste recognition and soldering: the solder paste is placed on ex vivo tissue (pig dura mater), one fiber is used for laser excitation, one fiber is used for fluorescence measurement, and a thermal camera is used as reference. The nanothermometry spectra measured by the spectrometer are used to control the feedback loop through an Arduino that modulates the laser power. f) Images show liver soldered with manual and power‐controlled methods. Manual soldering shows burnt areas, uneven bonding, and tissue dehydration, while power‐controlled soldering produces a uniform, damage‐free seam.

Aiming to attain increased precision and reliability in laser tissue soldering, the adoption of an automatic feedback control system emerges as an appealing choice. This preference arises from the capacity to eliminate user‐dependent variables, thereby improving the reliability and safety of the process. Thus, we implemented a Proportional‐Integral‐Derivative (PID) controller to maintain the temperature at the target site within the patient's body by regulating the laser power, driven by real‐time temperature measurements provided by fluorescent nanothermometry (**Figure** **2**). PID controllers are an established feedback control loop used in industrial applications, thanks to their ease of integration and built‐in stability. Their efficacy is further underscored by the tunability of three key parameters: proportional gain (P), integral gain (I), and derivative gain (D). These parameters allow for customization of controller responsiveness and speed, facilitating optimization for speed or the mitigation of overshooting, contingent on specific procedural needs. By optimizing the parameters, the controller can reliably maintain target temperatures in the range relevant to laser tissue soldering while minimizing rise times (below 10 s) and showing no overshooting (Figure 2a). For applications in MIS, performance under a variety of different application scenarios is essential for patient safety and outcome. Appropriate tailoring of the controller gives resilience to the system to vertical movements, reaching and maintaining the target temperature, albeit with variations in rise time when distances undergo significant changes (Figure 2b). Yet, nanothermometry reliably measures the temperature also at different distances. The parameters of the controller can further be tuned to make it more responsive, reaching the target temperature much faster and showing only a small overshoot (Figure 2c). The responsiveness also explains the initial high laser powers, with consequent high signal strength, that quickly goes to lower levels, due to the responsiveness of the PID controller.

Furthermore, we demonstrate that this technology succeeds when navigating diverse sections and complex topologies of a wound (Figure 2d,e). For demonstration, two pieces of solder paste 2 mm apart are placed on a piece of tissue, while the laser is moved at a constant speed (2 mm min^−1^) across them. The soldering temperature is maintained at the set target temperature of 60 °C throughout the entire section on the solder paste (green‐shaded area in Figure 2d). Upon detection of healthy tissue and areas devoid of solder material, the controller adjusts laser power to a secure level, averting potential damage that could result from inadvertent heating. Although NIR lasers generally have low absorption by tissue, conditions like high pigment concentration or proximity to blood‐rich areas, such as liver tissue,^[^ ^32^, ^33^, ^34^ ^]^ can lead to unintended localized heating even with NIR. Thus, the auto‐power reduction function is an additional safeguard that prevents such risks by reducing power in the absence of solder material.

This feature is enabled by the fluorescence emission signal from the paste. When signal strength is low, the laser power reduces to a safe level that still permits fluorescence detection (red‐shaded areas in Figure 2d), ensuring soldering resumes correctly once back over the paste (green‐shaded areas in Figure 2d). Additionally, reflected excitation light helps detect distance from the paste or proximity to tissue. At paste edges (yellow‐shaded areas in Figure 2d), temperatures may be overestimated due to a lower signal‐to‐noise ratio; however, the laser power is lowered, ensuring a safe process. To further demonstrate the advantages of controlled soldering over traditional manual tissue soldering, an additional experiment was conducted. A piece of solder paste was placed on a liver incision and soldered in a single pass from one end of the cut to the other, using either manual soldering or automated trajectory control with power modulation. Figure 2f illustrates the outcomes of this comparison. The first notable difference is in the uniformity of the soldered bond: controlled soldering produced a consistent, uniform bond with a constant width, while manual soldering resulted in variable bond width and visible burn marks, identifiable as darker brown spots. The second difference concerns the tissue integrity: in the manually soldered sample, thermal damage due to inaccurate laser positioning and dehydration of the surrounding tissue are clear. In contrast, controlled soldering yielded a uniform seam without signs of thermal damage from laser misalignment or tissue dehydration.

### 2.3 Machine Learning for Low‐Cost Thermal Distribution Imaging

During soldering, the temperature distribution provides valuable information for estimating the size of the heat‐affected area and how quickly previously heated areas are cooling down. Fluorescent nanothermometry requires detailed spectral information. However, a simple setup using only a fiber‐coupled laser and spectrometer lacks sensitivity to spatial differences. Temperature distribution information can be used to ensure a more uniform soldering throughout the entirety of the solder paste, as well as avoiding local hot spots that can damage the tissue. Spatially resolved 2D spectra can only be captured with specialized cameras like hyperspectral ones, which are relatively expensive. This applies to nanothermometry based on ratiometric methods with closely spaced peaks, as well as approaches involving peak shifts and fluorescence decay time.

**Figure 3:**
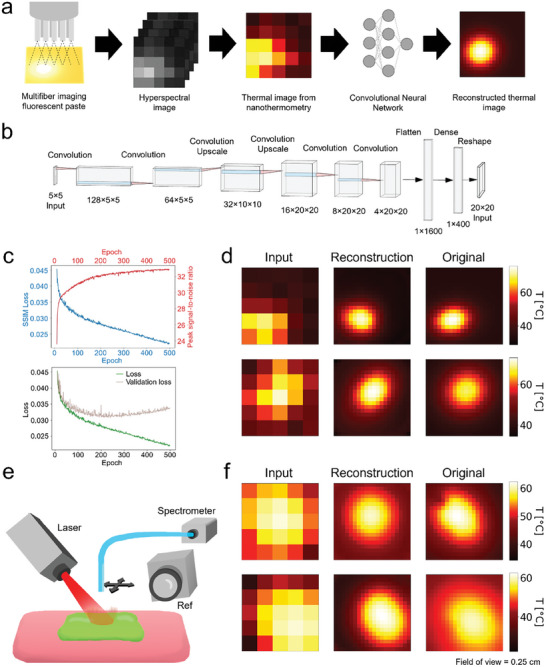
Machine learning approach for low‐cost thermal distribution imaging. a) Illustration of how a multifibre system coupled with the upscaling Convolutional Neural Network (CNN) can reconstruct the thermal distribution during laser excitation starting from a low‐resolution hyperspectral image. b) Upscaling CNN architecture. c) Performance of the trained CNN showed with training loss. d) Examples of simulated data reconstructed using the upscaling CNN. e) Experimental setup used to collect the fluorescence from each individual fiber featuring inexpensive equipment: the fluorescence light is collected by a displaceable fiber and is lead, after being filtered, to a spectrometer. The signal is used to calculate the temperature which is compared to a reference thermal camera. f) Examples of experimental data reconstructed using the upscaling CNN. (FOV = 0.25 cm).

In this context, we introduce a machine learning approach based on Convolutional Neural Networks (CNN) combined with a lens‐free, low‐resolution fiber bundle probe (**Figure** **3**) for thermal distribution measurement in MIS. Using a fiber bundle with a small number of fibers, such as a 5 × 5 square array, results in a low‐resolution hyperspectral image. These low‐resolution images can be challenging to interpret and do not offer significant advantages when presented to professionals performing the procedure. Furthermore, they tend to overestimate the temperature at the edges of the soldered area, primarily because the signal primarily originates from illuminated portions of the paste, which typically have higher temperatures. Our developed upscaling CNN, based on emerging architectures,^[^ ^35^ ^]^ can accurately reconstruct the thermal distribution and increase the resolution by a factor of 4. This substantial resolution improvement is achievable due to the limited diversity of temperature distribution profiles during laser irradiation. To train the CNN, we first generated a dataset by simulating various temperature distributions and their corresponding fluorescence signals detected by each fiber tip, including simulated noise. While Structural Similarity Index Measure (SSIM) loss and peak signal‐to‐noise ratio tend to improve with increasing epochs, the validation loss suggests that the optimal training performance occurs at ≈250–300 epochs. Therefore, it is advisable to select this range to avoid overfitting to the training data (Figure 3c). The CNN demonstrates strong performance in reconstructing the thermal distribution's position, size, and range (including peak temperature) within the training dataset, even when the input data contains limited information (Figure 3d). We further validate the CNN's performance using experimental data. A stage is employed to position a fiber tip where each fiber in a given bundle (in this case, a 5 × 5 square array of fibers side‐by‐side) would be located, allowing us to collect light at various positions. The fluorescence signal is then used to reconstruct the thermal distribution, which is compared to a reference thermal distribution obtained using a thermal camera (Figure 3f). Importantly, the thermal distributions calculated using the CNN match the measured distributions well and the peak temperatures are predicted with high accuracy (*ΔT_max_* = 2.6 ± 1.0 °C, SSIM = 0.85 ± 0.04, *N* = 3). This approach is also directly applicable to fluorescent nanothermometry based on peak shifting or lifetime measurements, where precise spectral information is required and provides a low‐cost route to thermal distribution imaging in MIS.

### 2.4 Thermal Distribution Imaging with Image‐Guiding Fiber and NIR Cameras in Robotic Soldering

**Figure 4:**
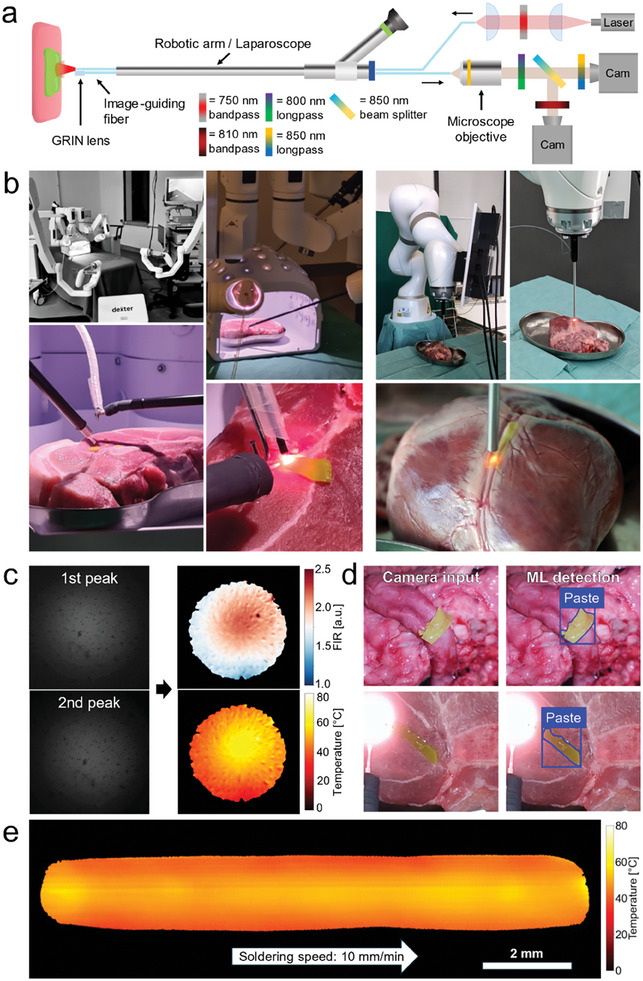
Multicore fiber approach for thermal imaging in robotic soldering. a) Schematic of the setup used to measure the thermal distribution during soldering, featuring two NIR cameras to detect the fluorescence of the two emission peaks. b) Pictures of the robotic systems with integrated tissue soldering equipment. Left side: telemanipulated robot (Dexter, Distalmotion SA); right side: programmable robot (LBR Med 14 820, KUKA). For the telemanipulated robot, the dual‐fiber system for automatized feedback‐controlled soldering is incorporated with the instrument of the left robotic arm, while the imaging fiber is spatially manipulated using a gripper instrument of the right robotic arm. For the programmable robot, a custom‐made attachment in which all the fibers are placed is used for the integration. c) Example of images recorded with the two different filters during soldering of a piece of solder paste. The FIR is calculated from the two images that represent the fluorescence of the two peaks (acq. time = 500 ms). The FIR is then used to compute the temperature distribution of the solder paste (FOV = 0.6 cm). d) A machine learning algorithm is developed and applied for the automatic detection and localization of the paste during surgery, information that can be used to automatically define and generate a soldering path for the robot. Top: image of the paste on a porcine aorta used for training. Bottom: picture taken by a laparoscope at the start of robotic soldering. e) The thermal imaging through fluorescence can be implemented together with the automatized feedback‐controlled soldering. This reconstructed image, made by adding images taken while the soldering probe was moving, shows how the temperature is kept at the target temperature (60 °C) throughout the entire soldering path.

Alternatively, instead of using the upscaling algorithm and spectrometer setup, image‐guiding fibers (also known as leached fibers) can be employed in combination with NIR cameras to obtain valuable thermal distribution information in a minimally invasive manner (**Figure** **4**). Image‐guiding fibers are thin bundles of thousands of fibers arranged orderly, allowing them to transmit images from one side of the fiber to the other with minimal signal loss and cross‐talk between fibers.^[^ ^36^ ^]^ To retrieve the temperature distribution, we use a dichroic beam splitter to separate the two emission peaks and two NIR cameras. A GRIN lens (gradient‐index lens) is used to create an image on the object side, while a microscope objective captures images on the distal side of the fiber with the two cameras (Figure 4a).

By adjusting the position of the GRIN lens relative to the fiber tip, we can change the focus and field of view (FOV). The choice of BiVO_4_:Nd^3+^ nanothermometer is particularly well‐suited to this method because its two thermally coupled emission peaks are spectrally distant (>40 nm apart) and can be clearly separated using commercially available dichroic beam splitters, bandpass, and longpass filters. Additionally, the used wavelengths fall within the sensitivity range of CCD and CMOS cameras, making it possible to utilize established and cost‐effective technology, as opposed to InGaAs or Mid‐IR image sensors.

This system can also be coupled with the automated feedback for temperature control and be readily applied to robotic surgery (Figure 4b). The integration is demonstrated with two distinct commercially available surgical robots with different control mechanisms. In the first case, the flexible and thin fibers are easily incorporated with the instrument of a teleoperated surgical robot (here exemplarily shown using Dexter, Distalmotion SA), and their position can be easily controlled through the remote robotic interface (Figure 4b, left). Alternatively, the system is used with a programmable surgical robot (LBR Med 14 820, KUKA). In this case, the fibers are passed inside a metal tube with an outer diameter of 5 mm with is attached to the end of the robot with a 3D printed attachment (Figure 4b, right). The small outer diameter is compatible with the use of smaller and less invasive trocars, as well as being the most common size of surgical laparoscopic instruments. The surgical workflow is as follows: First, the paste is deployed through one of the trocars and placed on the area of interest using the articulated robotic gripper, then, soldering is started by moving in place the instrument with the two fibers for the feedback‐controlled soldering. The image‐guiding fiber is inserted through another trocar and moved to the soldering site for monitoring purposes by the gripper arm of the surgical robot. During soldering, the thermal distribution can be seen thanks to the image‐guiding fibers. The two NIR cameras capture fluorescence images with the same acquisition time (0.5 s) but different gain values, given that the first peak (at 820 nm) is dimmer than the second peak (at 870 nm). Following a calibration obtained from reference measurements specific to this system and considering the gain values of the cameras, the FIR distribution can be converted into the thermal distribution of the paste (Figure 4c). Only temperature measurements from areas with sufficient signal strength are displayed.

Information about the spatial paste position can be given to the robotic system to enable soldering in an autonomous fashion, e.g., generating temperature‐optimal trajectories. To this aim, a machine learning image segmentation algorithm compatible with real‐time applications was developed. The solder paste detection was achieved by using this algorithm, able to reliably recognize and locate the solder paste even in images taken by the robotic laparoscope camera (Figure 4d, Supplementary Information). Such information can be used to automatically define a soldering path with the aim of soldering an extended region, such as the region along a cut or a suture line. Moreover, thanks to the automatic temperature control, the temperature can be kept at the desired value even while the soldering setup is moved along the soldering path in a fully automated manner.

This is demonstrated exemplarily by the temperature map acquired by the multifiber imaging system (Figure 4e), where the various temperature distributions acquired during soldering of a 13 mm‐long piece of solder paste are presented. Each frame can be linked with its location on the paste, allowing the creation of the temperature map of the paste during soldering. Such a map can be used to ensure optimal heating of the entire target area, avoiding local overheating spots or areas with suboptimal heating that would hinder proper tissue bonding, and can be fully integrated into the robotic setting. For example, robot control can be complemented by a cognitive supervisory framework^[^ ^37^ ^]^ to increase the level of task autonomy and facilitate uniform area heating at targeted temperatures realizing homogeneous tissue bonding.

### 2.5 Minimally Invasive Laser Tissue Soldering In Vivo

**Figure 5:**
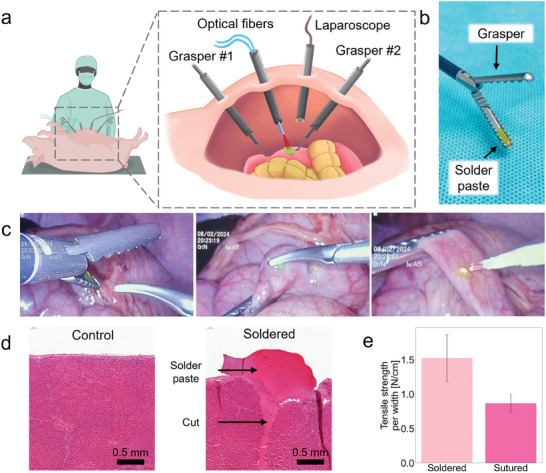
Application in laparoscopic surgery in vivo. a) Soldering performed laparoscopically on an in vivo porcine model, with the optical fibers passing through a trocar. b) The solder paste is placed in the groove of a grasper for easy insertion inside the abdominal cavity. c) Then, the paste can be taken and positioned with a second grasper. The optical fibers are directed to the paste and soldering is performed. d) Histology of the soldered liver over a surgical incision compared with a healthy control, showing no visible thermal damage and good adhesion. e) Tensile strength values of soldered liver compared to sutured liver, showing that soldering achieves higher bond strength due to its uniform adhesion across the tissue, which distributes stress more evenly and minimizes localized tearing common with sutures.

The potential of iSoldering for MIS is further demonstrated in an in vivo porcine model with torso dimensions approximating the ones of human patients (**Figure** **5**). The optical fibers for soldering can be easily incorporated into a typical laparoscopic setup by inserting them into a 5 mm trocar (Figure 5a). To insert the solder paste inside the abdomen, the solder paste can be placed in the groove of a grasper, without the need for other specialized equipment (Figure 5b). With this modality, the paste is protected by the grasper during insertion, allowing it to maintain its integrity throughout the insertion process even through narrow trocars. The paste is then taken using a second grasper and then positioned where needed, such as on a piece of intestine (Figure 5c). The intestine was chosen as it is one of the most challenging organs for laparoscopy.

The use of crosslinked GelMA instead of normal gelatine is of key importance in laparoscopy, as the working environment is kept at body temperature, causing the melting of non‐crosslinked gelatine‐based paste in less than a minute. By using crosslinked GelMA the paste does not melt and can be easily placed on the wound by the graspers. The optical fibers can be directed toward the area to solder by moving the trocar through which they are inserted and sliding them in or out to adjust the fiber‐tissue distance.

Feedback‐controlled soldering can be safely initiated when the fiber is approximately in place. The algorithm ensures that high laser powers are used only when the solder paste is detected (Figure 5c). When on the surrounding healthy tissue, the laser power is automatically reduced to avoid damage, while still providing useful information to guide the fiber positioning. The optical fibers can also be maneuvered with a grasper, allowing for precise illumination and soldering of harder‐to‐reach areas, such as damaged areas on the lateral abdominal wall (see Supporting Information). The effectiveness of feedback‐controlled iSoldering in MIS is further demonstrated based on histological data (Figure 5d), showing a surgical incision closed with strong adhesion and no visible tissue damage. Good adhesion is further demonstrated based tensile strength testing performed on porcine liver (lap shear configuration), achieving higher tensile strength than sutures (Figures 5e, and S4 and S6, Supporting Information). Traditional sutures rely on mechanical strength, often causing tearing in fragile tissues due to concentrated tension points. In contrast, laser soldering provides a continuous, uniform bond that distributes forces across the tissue, reducing the risk of tearing and significantly enhancing bond stability. Our study primarily focused on smaller wounds, but this soldering technique has no strict limitation on wound width, as larger wounds can be closed with additional soldering time. This approach, with its high bonding stability and reduced tissue strain, shows promising applications for tissues where traditional sutures may not be ideal, supporting its potential for future in vivo studies.

## 3 Conclusion

This work presents the integration of nanothermometry‐guided laser tissue soldering into the minimally invasive surgical procedures, addressing significant surgical limitations that have persisted hitherto. The inherent delicacy and complex structure of soft tissues often render conventional mechanical fusion techniques like suturing and stapling inadequate or impractical. The presented approach of laser tissue soldering, guided by real‐time fluorescent nanothermometry, emerges as a compelling solution to these challenges, opening new avenues for secure, precise, and controlled soft tissue fusion in MIS, including robotics. The solder paste primarily comprises albumin and gelatin, both biocompatible materials. The nanoparticles constitute only a minor fraction (0.002–0.3%) of the paste, and, as embedded components, their release after topical application remains limited.^[^ ^38^ ^]^ Such slow release is likely to show minimal or no adverse effects.^[^ ^39^, ^40^, ^41^ ^]^ Nonetheless, long‐term studies are planned to further assess safety and biocompatibility. By leveraging the precision and real‐time feedback offered by fluorescent nanothermometry, laser tissue soldering overcomes traditional barriers, presenting a straightforward, reliable, and adaptable methodology conducive to seamless incorporation within laparoscopic, endoscopic, and robotic surgical systems. This intricate thermal control, and its integration into robotic navigation mitigates the risks associated with overheating or underheating, thus substantially augmenting the safety and usability of the procedure. Additionally, the minimally invasive thermal imaging capability augments the visualization of the soldering process, offering surgeons an enhanced perspective and a higher degree of control, making this technology not only safe but highly user‐friendly. Moreover, the developed platform could potentially benefit the field of hyperthermia at large (for cancer and other pathologies), where precise light dosing is also crucial.

The research presented in this paper paves the way toward safe damage‐free automatized surgery on fragile internal tissues. After many years of research on laser‐based tissue bonding, the proposed approach is breaking the barrier that is preventing this technique from becoming widely accepted in clinical practice. Further investigation and development on data‐driven automation will allow for unprecedented wound closure reliability and reproducibility.

## 4 Experimental Section

### Paste Formulation

The solder paste was adapted based on the formulation reported in ref. [^[^ ^24^ ^]^], however, GelMA was added for improved properties during robotic manipulation and reduced fluidity at body temperature and during the soldering process. Unless specified, all concentration percentages are expressed as mass of solute over mass of solvent (water). Briefly, water solutions of bovine serum albumin (BSA, Sigma–Aldrich, A2153), gelatin (from porcine skin, gel strength 300, Type A – Sigma–Aldrich, G2500), flame‐made BiVO_4_:Nd^3+^ nanoparticles,^[^ ^29^ ^]^ and TiN nanoparticles (PlasmaChem GmbH, PL‐HK‐TiN) were mixed and then casted into the appropriate mold. The paste was cooled for at least 1 h at 4 °C and then kept at that temperature until use, not later than 2 weeks after production. The pastes that contained GelMA were produced using gelatin modified with methacryloyl (GelMA) instead of normal gelatin. The GelMA was synthetized following Pepelanova et al.^[^ ^42^ ^]^ A solution of 0.0069% LAP (Lithium phenyl‐2,4,6‐trimethylbenzoylphosphinate, Sigma–Aldrich, 900889) was used as crosslinking initiator and added to the solution as last component. The paste was first left for at least 1 h at 4 °C and then crosslinked under UV irradiation for 5 min (UVASPOT 400T, Dr. Hönle AG). Crosslinking of the paste prevents melting before BSA denaturation.

### Nanoparticle Characterization

The calibration of BiVO_4_:Nd^3+^ nanoparticles was carried out calculating the fluorescence intensity ratio of the two fluorescence peaks. The two peaks were defined as the regions between 790–840 nm and 840–945 nm respectively. Transmission electron microscopy (TEM) of BiVO_4_:Nd^3+^ and TiN nanoparticles was carried out using a transmission electron microscope (EM900, Carl Zeiss Microscopy GmbH) at 80 kV. Holey carbon‐coated copper grids (200 mesh, EM Resolutions) were incubated with poly‐ l ‐lysine solution (P8920, Sigma–Aldrich) for 10 min and subsequently washed with ultrapure (milliQ) water. The BiVO_4_:Nd^3+^ and TiN nanoparticles samples were first dispersed in milliQ water and subsequently drop‐casted onto the grids.

### Feedback Loop: Optical Setup

As excitation and heating source, a 750 nm CW laser (MDL‐III‐750‐2 W, CNI Lasers) with a maximum output power of 2 W was used. The power of the laser was controlled with pulse‐width‐modulation (PWM) via an Arduino microcontroller (Arduino UNO). A sturdy portable optical module was used to increase the monochromacity of the laser light. This module includes: an optical fiber (NA 0.22, ⌀400 µm) to guide the laser from the laser head to the optical module; a collimating plano‐convex lens (*f* = 30 mm, 780 nm V‐coating; Thorlabs); a 750 nm bandpass filter (FWHM = 40 nm; FB750‐40, Thorlabs); a plano‐convex lens (*f* = 30 mm, 780 nm V‐coating; Thorlabs) for coupling with the output fiber. A medical optical fiber (NA 0.37, ⌀600 µm, 1 m length; Wuhan Medfibers Technology Co.) was used to deliver the laser light to the target. After passing the optical module and the medical fiber the maximum available power was reduced to 1.22 W.

To record the fluorescence a NIR spectrometer (100 µm slit; STS‐NIR, Ocean Optics) was used. A medical optical fiber (NA 0.37, ⌀600 µm, 1 m length; Wuhan Medfibers Technology Co.) was used to collect the fluorescence light from the target. The medical fiber was connected to a sturdy optical module used to filter the fluorescence light. This module includes: a collimating achromatic doublet lens (*f* = 30 mm; Thorlabs); a 785 nm longpass filter (47‐508, Edmund Optics); an achromatic doublet lens (*f* = 30 mm; Thorlabs) for coupling with the fiber (NA 0.22, ⌀550 µm) connected to the spectrometer.

A thermal camera (PI 640i, Optris) was used to record reference data. Additionally, the tips of the fibers were fed through the aperture of a standard 5 mm trocar, to showcase the compatibility with common laparoscopic instruments. Apart from when otherwise stated, the same optical components (laser, spectrometer, filter and fibers) were also used in the other experiments.

### Feedback Loop: Controller

The controller algorithm was build based on the Python module ″simple‐pid v2.0.0″ by Martin Lundberg.^[^ ^43^ ^]^ A minimum laser power is set between 10 and 20%, depending on the configuration used, which is also used as starting laser power. The acquisition time is set between 300 and 800 ms.

To derive the calibration a data set consisting of fluorescence spectra and their corresponding temperatures is needed. The calibration can be performed either using a heating plate or using the laser itself as source of heat. Individual spectra were acquired in regular intervals and the reference data was taken with a thermal camera. The calibration was repeated with each paste sample for increased accuracy.

The general workflow is here presented:

1. The program measures a spectrum to correct for any background signal that is present. This step is repeated after a user‐defined number of iterations (default: 100 iterations).
2. The laser is started with a pre‐specified minimum power (default: 20%). The minimum power is chosen depending on the working distance and heating characteristics of the paste. It can be reduced when the temperature keeps on rising at minimum power, while it may be raised to increase fluorescence signal, such as at long working distances.
3. The spectrum is taken, with a user‐defined integration time (default: 500 ms). The integration time may be adjusted based on signal strength, with lower values yielding better controller response at the cost of SNR. The integration time is the main limitation of the loop sample rate.
4. If the number of counts is too low (default: 150 peak counts), the laser is switched to the minimum power to avoid heating healthy tissue.
5. The temperature is calculated and fed into the PID regulator, returning a new value for the laser power. The PID gain parameters can be adjusted during run time, with presets available.

The PID controller was tuned heuristically, with the following main performance requirements, in order of importance: stability in all scenarios; overshoot lower than 5 °C; *T*_90_ (time to rise to 90% of the set value) lower than 20 s. Setpoint‐tracking was deemed secondary since the target temperature will most likely be held constant in LTS operation, putting the focus instead on disturbance rejection (movement during soldering).

### Feedback Loop: Soldering

The performance of the system was evaluated in different scenarios. In all cases, a solder paste with 25% BSA, 6.5% Gelatin, 0.3% BiVO_4_:Nd^3+^, and 0.002% TiN was used. The solder pastes were placed on top of a piece of ex vivo porcine dura mater (retrieved from the local slaughterhouse), which was placed on a moving stage (a modified 3D printer). The distance of the sample from the fiber tip was changed between 0.5 and 1.5 cm, while the target temperature was changed between 50 and 80 °C. In the case of the moving soldering, the sample was moved at the speed of 2 mm min^−1^.

In the comparison between manual soldering and controlled soldering, the solder paste was placed on a surgical incision of a porcine liver.

### CNN Training Dataset Creation

The process used to generate simulated data to train the CNN for the reconstruction of temperature distributions in a luminescence nanothermometry temperature profile is here explained. The training data must have the measured thermal images (i.e., 5 × 5 images) and the respective original 640 × 480 thermal images. A simulation algorithm that simulates the fluorescence received by an optical fiber placed in front of a laser‐excited surface with BiVO_4_:Nd^3+^ nanoparticles was developed and used. The simulation algorithm for data creation was divided into three parts: database creation, modeling, and image formation.

In the first step, the algorithm was building a temperature‐spectrum database. Using a thermally controlled cell (qpod 2e, Quantum Northwest), a 0.5% BiVO_4_:Nd^3+^ water solution in a quartz cuvette was excited by a 750 nm laser with a 750 nm bandpass filter at various temperatures. The solution was stirred using a magnet at 1400 rpm. The spectra were measured using a spectrometer with a 785 nm longpass filter, placed perpendicularly to the laser beam. Experimentally measured spectra were correlated with temperatures measured by a type K NiCr‐Ni thermocouple placed in the water dispersion. Temperatures were changed with steps of 10 °C between 30 and 80 °C. Assuming a linear approximation, the spectra data points can be expanded to form a complete temperature‐spectra database for all temperatures between 30 and 80 °C.

The modeling stage includes models for the laser excitation, fiber acceptance region, and temperature distribution. The fluorescent surface is divided into *N* = 640 × 480 pixels. The spectrum recorded by a fiber with a specific NA, placed in specific *xyz* coordinates is approximated by the following equation:

(1) $$I_{xyz}\;\left(\lambda \right)=\sum_{i}^{N}I_{i}\left(\lambda ,T_{i}\right)\cdot P_{i}\cdot F_{xyz,\;i}\cdot c_{i}$$

where *I_xyz_* (λ) is the fluorescence intensity at each wavelength *λ* measured by fiber in position *xyz*, *i* is the index corresponding to each of the *N* pixels, *I_i_* (λ,*T_i_*) is the fluorescence emitted from the pixel *i* at the temperature *T_i_*, *P_i_* is the laser power intensity, *F*_*xyz*, *i*_ is the fraction of fluorescence emitted by the pixel *i* that is measured by the fiber in position *xyz* (which takes into account acceptance angle of the fiber and distance from source), and *c_i_* is the concentration of nanothermometers in the pixel *i*. All parameters are normalized and range from 0 to 1. The laser power distribution is calculated using the paraxial Helmholtz equation for TEM_00_ Gaussian and top‐hat beams.

In the image formation step the algorithm is run multiple times varying the fiber position with fixed laser, temperature and concentration parameters to create the simulated measured image. A 5 × 5 grid with 500 µm spacing was chosen for the simulation. Moreover, a laser placed in the center of the FOV with beam quality factor *m* = 8, at a distance of 10 mm and beam waist of 1.5 mm was simulated. The nanothermometer concentration was assumed constant and the fiber dimensions were set to NA 0.22 fiber, core diameter 200 µm, distance of 1 mm away from the solder.

In order to produce the training setup, a set of various temperature distributions were created. Skewed Gaussian profiles with different temperature range, position, angle, and width were used due to their similarity to temperature profiles found during laser irradiation. The simulation algorithm was then run for each different temperature profile. The set of values presented in Table 1 was used to create the temperature distributions. The following parameters were varied: the peak position *µ_x_* and *µ_y_*, the two standard deviations *σ_x_* and *σ_y_*, the skewness *a_x_* and *a_y_*, the maximum and minimum temperatures *T*_max_ and *T*_min_, and the rotation *φ*. *T*_min_ was chosen to be randomly assigned in the interval [20, *T*_max_ – 1] with uniform distribution.

**Table 1:** Simulation parameters.

	*µ_x_, µ_y_* [mm]	*σ_x_, σ_y_* [10^−4^ mm]	*a_x_*	*a_y_*	*T*_max_ [°C]	*φ* [°]
Values	−1 to 1	5–11	−2 to 2	0	40–90	0–90
Step size	0.5	2	4	0	5	15

The dataset was further improved by discarding samples with low degree of similarity, choosing to use a Structural Similarity Index Measurement (SSIM) of 0.7 as cut‐off value. The threshold of 0.7 was carefully selected, representing an optimal balance between preserving data quality and maintaining enough data for training. This approach led to the removal of 10′010 samples out of a total of 38′947, leading to a remaining 28′937 samples, which provide ample data for effective network training.

### CNN Architecture and Training

The chosen CNN architecture is shown in Figure 3b. Comprised of several convolutional layers, the model integrates upsampling layers between the second and third, and the third and fourth layers. These upsampling layers amplify the image size by a factor of two each time. This action results in an enhancement of resolution, a pivotal step in image upscaling tasks. The activation function selected for this model is the PReLU (Parametric Rectified Linear Unit), a variant of the leaky ReLU equipped with a trainable parameter. This added flexibility enables the PReLU function to adapt its slope during training, allowing the network to discern where non‐linearity should be introduced. In terms of loss function, the SSIM (Structural Similarity Index Measure) loss has been favored over the MSE (Mean Squared Error). The motivation behind this choice is the superior performance of SSIM in preserving structures and shapes, which is crucial for the objectives. Serving as the cornerstone of this Convolutional Neural Network (CNN), the convolutional layers are endowed with a set of learnable filters, often referred to as kernels. As the forward pass is carried out, each filter sweeps over the width and height of the input volume, performing a dot product operation between the entries of the filter and the input. This process generates an activation map. Importantly, each layer was tailored to detect different features within the image, with earlier layers often picking up rudimentary features such as edges, while subsequent layers may capture more complex features like textures. During the training phase of the CNN, a configuration of 300 epochs with a batch size of 500 and a learning rate set to the conventional value of 0.001 were used. To optimize the network architecture, the training dataset was divided as follows: a quarter of the entire dataset was reserved for testing, while the remaining 75% was split further into an 80‐20 ratio for training and validation, respectively. Following the identification and optimization of the most promising network configuration, it was subjected to an additional round of training involving the complete artificially generated dataset. This comprehensive retraining was executed to enhance the performance of the network when dealing with data derived from laboratory tests.

### CNN Experimental Validation

A repurposed 3D printer (Ender 2 Pro, Creality) was used as XYZ translation stage with temperature control. A solder paste sample (25% BSA, 6.5% GelMA, 0.3% BiVO_4_:Nd^3+^, 0.002% TiN, 0.0069% LAP) was placed on the platform of the XYZ translation stage and underwent heating via laser irradiation. The 750 nm laser, filtered by a 750 nm bandpass filter and focused by a lens, was fixed on the platform and therefore did not move with respect to the paste. The aim of this setup is to mimic the conditions of any multicore fiber, such as one comprising 25 cores, arranged in a quadratic 5 × 5 configuration. An optical fiber (NA 0.22, ⌀600 µm) interfaced with the spectrometer was placed where the extruder of the 3D printer is usually placed. A 785 nm longpass filter was used to filter the collected radiation.

The measurement phase commences after the sample has been irradiated long enough to reach a stable temperature distribution. A picture with a thermal camera was taken at the beginning and end of the measurement. The stage is used to move the fiber through the 5 × 5 grid with 500 µm distance between points. The acquisition time of the spectrometer, usually 500 ms, was varied depending on the intensity of the fluorescence, reaching up to 6 s in extreme cases, when the fiber was particularly far from the excited region. The stage bed was also heated to circa 37 °C to simulate the temperature found in the body. The distance between the optical fiber and the sample was kept at ≈2 mm. The data was then processed to form the 5 × 5 low resolution thermal image. The trained CNN was used to reconstruct the thermal distribution and the result was compared to the resized data acquired with the thermal camera. *N* = 3 for experimental paste samples.

### Thermal Imaging through Image‐Guiding Fiber

A 54 cm long glass fiber optic image conduit (1534320, SCHOTT) was used to relay the fluorescence image from the solder paste to the NIR cameras. The image conduit has an outer diameter of 1.05 mm and 18k fibers. A medical fiber together with a 750 nm laser were used for excitation. A GRIN (Gradient Index) rod lens (working distance = 0 mm, length = 4.34 mm, diameter = 1.80 mm; #64‐525, Edmund Optics) was placed in front of the fiber and moved manually to the position necessary to focus the image. A x10 microscope objective (Bresser) was used to image the other end of the image conduit. A custom 3D‐printed filter cube was used to filter out the fluorescence signal from the laser light, as well as to separate the two fluorescence peaks. The following filters were used: 800 nm longpass filter (FELH0800, Thorlabs), 830 nm longpass beamsplitter (F38‐830, AHF Analysentechnik), 810 nm bandpass filter (FBH810‐10, Thorlabs), 850 nm longpass filter (FELH0850, Thorlabs). For the positioning of the filters see Figure 4a. Two NIR cameras (acA1920‐40 µm, Basler) were used to record the fluorescence of the two fluorescence peaks. A thermal camera was used as control. A piece of solder paste (25% BSA, 6.5% Gelatin, 0.3% BiVO_4_:Nd^3+^, 0.002% TiN) on a glass slide was used. The solder paste was placed at a distance between 0.3 and 1 cm away from the GRIN lens. The piece of solder paste was moved manually to simulate the movement of the fiber with respect to the paste during soldering. The laser power was changed between 30 and 80% of the maximum power to verify the performance of the imaging method. The acquisition time was set at 500 ms. The gains were set carefully to avoid saturation and achieve high signal‐to‐noise ratio. The fluorescence intensity ratios (FIRs) were computed after manually aligning the two images and after removing the background from the images, calculated using the average in a selected area of the images without signal. A Gaussian blur of standard deviation 7 pixels was added to avoid artifacts coming from dark fibers and imperfect image alignment. Moreover, only FIRs from pixels with more than 15 counts in the 810 nm peak image are displayed (all the others are set to 0 by default, appearing dark). The same has been done for thermal images. The calibration was computed through a reference measurement. Although the system features a beam splitter and a bandpass filter, the calibration would also take into account any inefficiency in separating the two peaks and any possible influence by the 1064 nm peak – which, however, has the same temperature dependence as the 870 nm peak and is only weakly detected by the camera sensors (QE < 2% above 1000 nm).

The imaging of the thermal distribution was also recorded during soldering of an extender region of the paste. The paste was moved using the *xyz* stage at a speed of 10 mm min^−1^ while soldering and imaging were performed. The composite image of the thermal distribution throughout the paste during soldering was created by computing the average temperature at each physical location whenever the temperature at that location was measured and deemed accurate (i.e., in the range 20–80 °C).

### Solder Paste Recognition ML Algorithm

An image segmentation model was trained on a dataset of diverse images of solder pastes of various shapes (up to squares of 1 cm^2^) and nanoparticles concentrations (0.3% BiVO_4_:Nd^3+^, 0.0002‐0.002% TiN) placed on various tissues, such as ex vivo and in vivo porcine blood vessels and intestine. A total of 150 pictures were used, with an 80/20 training/validation split. The images were taken with a smartphone, downscaled to 1920 × 1440 pixels, and then labeled manually. The training was performed on a pretrained model (″yolov8n‐seg.pt″ by YOLOv8.0.20, Ultralytics), with 25 epochs, image size of 640, and batch size of 10. More information can be found in Supplementary Information.

### Robotic Soldering

Two different robots were integrated with the soldering setup. In the first case, a surgical robot (Dexter, Distalmotion SA) controlled through a remote telemanipulator was used. The test was performed inside a laparoscopic trainer simulator box. The optical components used for the temperature‐controlled soldering and for the nanothermometry thermal imaging are the ones described in the previous sections. The fibers for the temperature‐controlled soldering were integrated with one of the surgical instruments. The image‐guiding fiber was inserted using a plastic sleeve through a trocar and moved in position using the robotic arm equipped with a grasper. A metal hook passed through the plastic sleeve aided the movement of the fiber and avoided damage to the fiber from direct grasping. A piece of solder paste was inserted and moved to the desired location using the robotic grasper. A piece of porcine tissue (pig shoulder) was purchased from a local grocery shop and used as model for the experiment. Soldering was then performed and nanothermometry thermal distribution data acquired.

In the second case, a programmable surgical robot (LBR Med, KUKA) was used. The attachment for the soldering setup integration comprises three parts: a stainless steel pipe (outer diameter of 5 mm, length of 20 cm) was used to contain all the fibers; a PETG 3D printed mount for attachment to the end side of the robot; a flexible resin 3D printed fiber tip attachment for correct and reproducible fiber positioning. Side holes were drilled into the steel pipe to allow fiber insertion into the tubes, as well as fixing of the tube on the 3D printed mount. A porcine heart was used as model for the experiments.

### In Vivo Laser Tissue Soldering

An in vivo study was approved by the Commission of Work with Experimental Animals (project ID: MSMT‐17853/2023‐5) under the Czech Republic's Ministry of Agriculture supervision. All procedures were carried out according to Czech regulations as well as those of the European Union. A combined intramuscular injection of ketamine (Narkamon 100 mg mL−1, BioVeta a.s., Ivanovice na Hané, Czech Republic) and azaperone (Stresnil 40 mg mL−1, Elanco AH, Prague, Czech Republic) was used to pre‐medicate a healthy female Prestice Black‐Pied pig (60 months old). Continuous intravenous propofol injection (Propofol 2% MCT/LCT Fresenius Medical Care a.s.) was used to maintain general anesthesia. Nalbuphine (Nalbuphin, Torrex Chiesi CZ s.r.o., Czech Republic) was used intravenously for analgesia assurance. The abdominal cavity was entered following ordinary laparoscopic procedure. The solder paste was cut and placed in a grasper to insert it inside the abdominal cavity. Optical fibers were passed through a 5 mm trocar and moved in position by hand. The intestine and the left lateral abdominal wall (where a defect was made) were soldered using the iSoldering approach, testing pastes of various composition (gelatine‐ or crosslinked GelMA‐based and TiN concentrations of 0.001%, 0.002%, and 0.01%). After euthanasia, tissue samples were collected and chemically fixed in 4% paraformaldehyde in PBS. Histology samples were processed (embedded, sectioned, and stained) by SophistoLab, Muttenz, Switzerland. Samples were imaged using a whole slide scanner at ScopeM, ETH Zurich.

### Mechanical Testing

Porcine liver was obtained from a local slaughterhouse and used the same day. The tensile strength was measured using a force meter (AFM‐20, TopHomer). Liver samples were cut into rectangular pieces (0.5 ± 0.1 cm width, 0.5 ± 0.1 cm height, 4.0 ± 0.5 cm length), with a full‐width cut placed in the middle. Soldered samples were soldered with a 0.5 × 0.5 cm square of GelMA‐based solder paste containing a TiN concentration of 0.004%, using a 750 nm laser for 1 min at 70 °C. Sutured samples were sutured using a 4/0 Poly(*p*‐dioxanone) absorbable suture (Polydox monofilament, Vitrex Medical A/S) with one knot. Failure was defined by the full separation of the specimen. Tensile strength was obtained from the breaking load normalized by the width of the specimens. *N* = 3 for both samples.

### Statistical Analysis

No data preprocessing has been employed. Data in bar and line charts are displayed as mean ± SD. The sample size (N) was indicated in the figure captions and Experimental Section. Python has been used for data processing.

## Conflict of Interest

O.C. and I.K.H. declare inventorship on a patent application by ETH Zurich and Empa: Composition for Laser Tissue Soldering, EP21216014.7. All other authors declare no conflict of interest.

## Author Contributions

O.C. and I.K.H. conceptualized the idea for the study. O.C., I.K.H., G.M., A.J., S.S., J.R., and D.K. designed the methodology for the study. O.C., T.L,, M.Z., M.S., G.M., S.A., C.S., S.S., and D.K. performed investigation. O.C. and I.K.H. performed the visualization. I.K.H. performed funding acquisition. I.K.H. performed project administration. O.C. and I.K.H. performed the supervision. O.C. and I.K.H. wrote the original draft. O.C., I.K.H., G.M., A.J., S.S., J.R., V.L., D.K., R.Q., T.L., M.Z., S.A., and C.S. wrote, reviewed, and edited the manuscript.

## Supporting information



Supporting Information

## Data Availability

The data that support the findings of this study are available from the corresponding author upon reasonable request.

## References

[1] K. Bingmer , A. Ofshteyn , S. L. Stein , J. M. Marks , E. Steinhagen , Surg. Endosc. 2020, 34, 967. 31183795 10.1007/s00464-019-06881-0

[2] S. Lim , S. Ghosh , P. Niklewski , S. Roy , JSLS 2017, 21, 201700021. 10.4293/JSLS.2017.00021 PMC5491803 28694682

[3] N. Fuchs Weizman , R. Maurer , J. I. Einarsson , A. F. Vitonis , S. L. Cohen , J. Surg. Edu. 2015, 72, 985. 10.1016/j.jsurg.2015.04.001 26143517

[4] H. Saeidi , J. D. Opfermann , M. Kam , S. Wei , S. Leonard , M. H. Hsieh , J. U. Kang , A. Krieger , Sci. Rob. 2022, 7, eabj2908. 10.1126/scirobotics.abj2908 PMC8992572 35080901

[5] J. Troccaz , G. Dagnino , G.‐Z. Yang , Annu. Rev. Biomed. Eng. 2019, 21, 193. 30822100 10.1146/annurev-bioeng-060418-052502

[6] P. E. Dupont , B. J. Nelson , M. Goldfarb , B. Hannaford , A. Menciassi , M. K. O'Malley , N. Simaan , P. Valdastri , G.‐Z. Yang , Sci. Robot. 2021, 6, eabi8017. 34757801 10.1126/scirobotics.abi8017 PMC8890492

[7] A. Meddahi‐Pellé , A. Legrand , A. Marcellan , L. Louedec , D. Letourneur , L. Leibler , Angew. Chem., Int. Ed. 2014, 53, 6369. 10.1002/anie.201401043 PMC4320763 24740730

[8] S. E. Tevis , G. D. Kennedy , J. Surg. Res. 2013, 181, 106. 23465392 10.1016/j.jss.2013.01.032 PMC3637983

[9] W. J. Halabi , C. Y. Kang , M. D. Jafari , V. Q. Nguyen , J. C. Carmichael , S. Mills , M. J. Stamos , A. Pigazzi , World J. Surg. 2013, 37, 2782. 23564216 10.1007/s00268-013-2024-7

[10] S. Basov , A. Milstein , E. Sulimani , M. Platkov , E. Peretz , M. Rattunde , J. Wagner , U. Netz , A. Katzir , I. Nisky , Biomed. Opt. Express 2018, 9, 5635. 30460151 10.1364/BOE.9.005635 PMC6238920

[11] J. Wu , H. Yuk , T. L. Sarrafian , C. F. Guo , L. G. Griffiths , C. S. Nabzdyk , X. Zhao , Sci. Transl. Med. 2022, 14, eabh2857. 35108064 10.1126/scitranslmed.abh2857

[12] A. H. C. Anthis , A. A. Schlegel , M. Hartel , I. K. Herrmann , Nat. Biomed. Eng. 2022, 6, 1089. 35760950 10.1038/s41551-022-00900-7

[13] A. P. Duarte , J. F. Coelho , J. C. Bordado , M. T. Cidade , M. H. Gil , Prog. Polym. Sci. 2012, 37, 1031.

[14] K. Zheng , Q. Gu , D. Zhou , M. Zhou , L. Zhang , Smart Mater. Med. 2022, 3, 41.

[15] D. M. Toriumi , W. F. Raslan , M. Friedman , M. E. Tardy , Archives Otolaryngol.–Head Neck Surg. 1990, 116, 546. 10.1001/archotol.1990.01870050046004 2183824

[16] Y. Takegawa , T. Takao , H. Sakaguchi , T. Nakai , K. Takeo , Y. Morita , T. Toyonaga , Y. Kodama , Sci. Rep. 2022, 12, 6986. 35484272 10.1038/s41598-022-10968-5 PMC9050883

[17] H. Yuk , C. E. Varela , C. S. Nabzdyk , X. Mao , R. F. Padera , E. T. Roche , X. Zhao , Nature 2019, 575, 169. 31666696 10.1038/s41586-019-1710-5

[18] I. Ashbell , N. Agam , A. Katzir , S. Basov , M. Platkov , I. Avital , I. Nisky , U. Netz , Heliyon 2023, 9, e16018. 37205994 10.1016/j.heliyon.2023.e16018 PMC10189270

[19] P. Matteini , F. Rossi , F. Ratto , R. Pini , in Laser Imaging and Manipulation in Cell Biology, John Wiley & Sons, Hoboken, NJ 2010, pp. 203–231.

[20] R. Schober , F. Ulrich , T. Sander , H. Dürselen , S. Hessel , Science 1986, 232, 1421. 3715454 10.1126/science.3715454

[21] D. Simhon , M. Halpern , T. Brosh , T. Vasilyev , A. Ravid , T. Tennenbaum , Z. Nevo , A. Katzir , Ann. Surg. 2007, 245, 206. 17245173 10.1097/01.sla.0000232554.13719.10 PMC1876995

[22] D. F. Gomes , I. Galvão , M. A. R. Loja , Appl. Sci. 2019, 9, 2157.

[23] D. Ghosh , C. M. Salinas , S. Pallod , J. Roberts , I. R. S. Makin , J. R. Yaron , R. S. Witte , K. Rege , Bioeng. Transl. Med. 2023, 8, e10412. 36925709 10.1002/btm2.10412 PMC10013809

[24] A. J. Kirsch , C. S. Cooper , J. Gatti , H. C. Scherz , D. A. Canning , S. A. Zderic , H. M. Snyder , J. Urol. 2001, 165, 574. 11176439 10.1097/00005392-200102000-00073

[25] Y. A. Mistry , S. S. Natarajan , S. A. Ahuja , Ann. Maxillofac. Surg. 2018, 8, 35. 29963422 10.4103/ams.ams_147_17 PMC6018299

[26] H.‐C. Huang , C. R. Walker , A. Nanda , K. Rege , ACS Nano 2013, 7, 2988. 23530530 10.1021/nn303202k

[27] P. A. York , R. Peña , D. Kent , R. J. Wood , Sci. Rob. 2021, 6, eabd5476. 10.1126/scirobotics.abd5476 34043580

[28] M. E. M. K. Abdelaziz , J. Zhao , B. Gil Rosa , H.‐T. Lee , D. Simon , K. Vyas , B. Li , H. Koguna , Y. Li , A. A. Demircali , H. Uvet , G. Gencoglan , A. Akcay , M. Elriedy , J. Kinross , R. Dasgupta , Z. Takats , E. Yeatman , G.‐Z. Yang , B. Temelkuran , Sci. Adv. 2024, 10, eadj1984. 38241380 10.1126/sciadv.adj1984 PMC10798568

[29] O. Cipolato , L. Dosnon , J. Rosendorf , S. Sarcevic , M. Zäch , A. Bondi , V. Liska , A. A. Schlegel , I. K. Herrmann , Small Methods 2023, 7, 2300693. 10.1002/smtd.202300693 37592160

[30] Z. Wu , F. Zeng , L. Zhang , S. Zhao , L. Wu , H. Qin , D. Xing , Nano Res. 2021, 14, 2717.

[31] G. M. Hale , M. R. Querry , Appl. Opt. 1973, 12, 555. 20125343 10.1364/AO.12.000555

[32] J. L. Sandell , T. C. Zhu , J. Biophotonics 2011, 4, 773. 22167862 10.1002/jbio.201100062 PMC3321368

[33] V. Dremin , E. Potapova , E. Zherebtsov , K. Kandurova , V. Shupletsov , A. Alekseyev , A. Mamoshin , A. Dunaev , Sci. Rep. 2020, 10, 14200. 32848190 10.1038/s41598-020-71089-5 PMC7449966

[34] A. N. Bashkatov , E. A. Genina , V. I. Kochubey , V. S. Rubtsov , E. A. Kolesnikova , V. V. Tuchin , Quantum Electron 2014, 44, 779.

[35] J. Kim , J. K. Lee , K. M. Lee , in 2016 IEEE Conf. on Computer Vision and Pattern Recognition (CVPR) , Las Vegas, NV, USA 2016, pp. 1646–1654.

[36] R. Nißler , E. Totter , S. G. Walter , J. T. Metternich , O. Cipolato , D. Nowack , A. Gogos , I. K. Herrmann , Adv. Healthcare Mater. 2024, 13, 2302950. 10.1002/adhm.202302950 PMC11481054 38245823

[37] D. Pardo , L. Fichera , D. Caldwell , L. S. Mattos , Neural Process Lett. 2015, 42, 55.

[38] M. T. Matter , J.‐H. Li , I. Lese , C. Schreiner , L. Bernard , O. Scholder , J. Hubeli , K. Keevend , E. Tsolaki , E. Bertero , S. Bertazzo , R. Zboray , R. Olariu , M. A. Constantinescu , R. Figi , I. K. Herrmann , Adv. Sci. 2020, 7, 2000912. 10.1002/advs.202000912 PMC7404155 32775166

[39] M.‐A. Shahbazi , L. Faghfouri , M. P. A. Ferreira , P. Figueiredo , H. Maleki , F. Sefat , J. Hirvonen , H. A. Santos , Chem. Soc. Rev. 2020, 49, 1253. 31998912 10.1039/c9cs00283a

[40] A. Sukhanova , S. Bozrova , P. Sokolov , M. Berestovoy , A. Karaulov , I. Nabiev , Nanoscale Res. Lett. 2018, 13, 44. 29417375 10.1186/s11671-018-2457-x PMC5803171

[41] F. H. L. Starsich , P. Gschwend , A. Sergeyev , R. Grange , S. E. Pratsinis , Chem. Mater. 2017, 29, 8158.

[42] I. Pepelanova , K. Kruppa , T. Scheper , A. Lavrentieva , Bioengineering 2018, 5, 55. 30022000 10.3390/bioengineering5030055 PMC6165498

[43] M. Lundberg , simple‐pid, version 2.0.0, according to Czech regulations as well 2023, https://github.com/m‐lundberg/simple‐pid (accessed: January 2024).

