# Supplementary material for: Robotic Laser Tissue Soldering for Atraumatic Soft Tissue Fusion Guided by Fluorescent Nanothermometry
Source: Adv Sci (Weinh). 2024 Nov 21;12(7):2406671. doi: 10.1002/advs.202406671 (PMC11831491; doi:10.1002/advs.202406671)
Supplement: Supplementary file 1 — Supporting Information [file ADVS-12-2406671-s001.pdf]

ADVANCED SCIENCE Open Access

Supporting Information

for *Adv. Sci.*, DOI 10.1002/advs.202406671

Robotic Laser Tissue Soldering for Atraumatic Soft Tissue Fusion Guided by Fluorescent Nanothermometry

*Oscar Cipolato, Tobias Leuthold, Marius Zäch, Georg Männel, Sam Aegerter, Calinda Sciascia, Alexander Jessernig, Marco von Salis, Sima Sarcevic, Jachym Rosendorf, Vaclav Liska, Dennis Kundrat, Romain Quidant and Inge K. Herrmann**

Supplementary Information

"Robotic Laser Tissue Soldering for Atraumatic Soft Tissue Fusion Guided by Fluorescent Nanothermometry"

*Oscar Cipolato,[1-4] Tobias Leuthold,[1] Marius Zäch,[1] Georg Maennel,[5] Sam Aegerter,[1] Calinda Sciascia,[1] Alexander Jessernig,[1-4] Marco von Salis,[1] Sima Sarcevic,[6,7] Jachym Rosendorf,[6,7] Vaclav Liska,[6,7] Dennis Kundrat,[5] Romain Quidant,[8] Inge K. Herrmann[1-4]**

[1] Nanoparticle Systems Engineering Laboratory, Institute of Energy and Process Engineering (IEPE), Department of Mechanical and Process Engineering (D-MAVT), ETH Zurich, Sonneggstrasse 3, 8092 Zurich, Switzerland.
[2] Particles Biology Interactions Laboratory, Department of Materials Meet Life, Swiss Federal Laboratories for Materials Science and Technology (Empa), Lerchenfeldstrasse 5, 9014 St. Gallen, Switzerland.
[3] The Ingenuity Lab, University Hospital Balgrist, Balgrist Campus, Forchstrasse 340, 8008 Zurich, Switzerland.
[4] University of Zurich, Faculty of Medicine, Rämistrasse 71, 8006 Zurich, Switzerland.
[5] Fraunhofer Research Institution for Individualized and Cell-Based Medical Engineering IMTE, 23562 Lübeck, Germany.
[6] Department of Surgery, Faculty of Medicine in Pilsen, Charles University, Alej Svobody 923/80, Pilsen, 32300 Czech Republic.
[7] Biomedical Center, Faculty of Medicine in Pilsen, Charles University, Alej Svobody 1655/76, Pilsen, 32300 Czech Republic.
[8] Nanophotonic Systems Laboratory, Institute of Energy and Process Engineering (IEPE), Department of Mechanical and Process Engineering (D-MAVT), ETH Zurich, Sonneggstrasse 3, 8092 Zurich, Switzerland.
*ingeh@ethz.ch; inge.herrmann@uzh.ch; inge.herrmann@empa.ch; +41 58 765 7153

Nanoparticle characterization

Transmission electron microscopy (TEM) images of BiVO and TiN nanoparticles are shown in Figure S1. TEM was performed using an EM900 (Carl Zeiss Microscopy GmbH) at 80 kV. Holey carbon-coated copper grids (200 mesh, EM Resolutions) were pre-treated by incubating with a poly-L-lysine solution (P8920, Sigma-Aldrich) for 10 minutes, followed by rinsing with ultrapure (MilliQ) water. The nanoparticle samples were then dispersed in MilliQ water and drop-cast onto the treated grids.

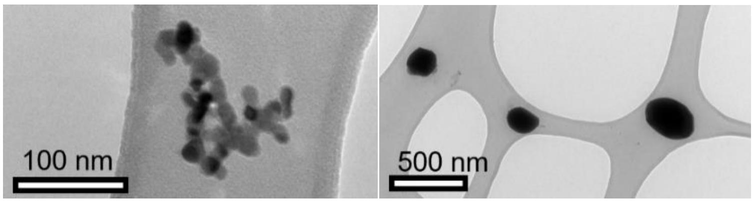

Figure S1: TEM images. *TEM images of TiN (top) and Nd-doped BiVO (bottom).*

Machine Learning paste detection from image

The training metrics of the machine vision algorithm are shown in Figure S2a. The performance of the algorithm is further demonstrated through additional images used for validation (Figure S2b).

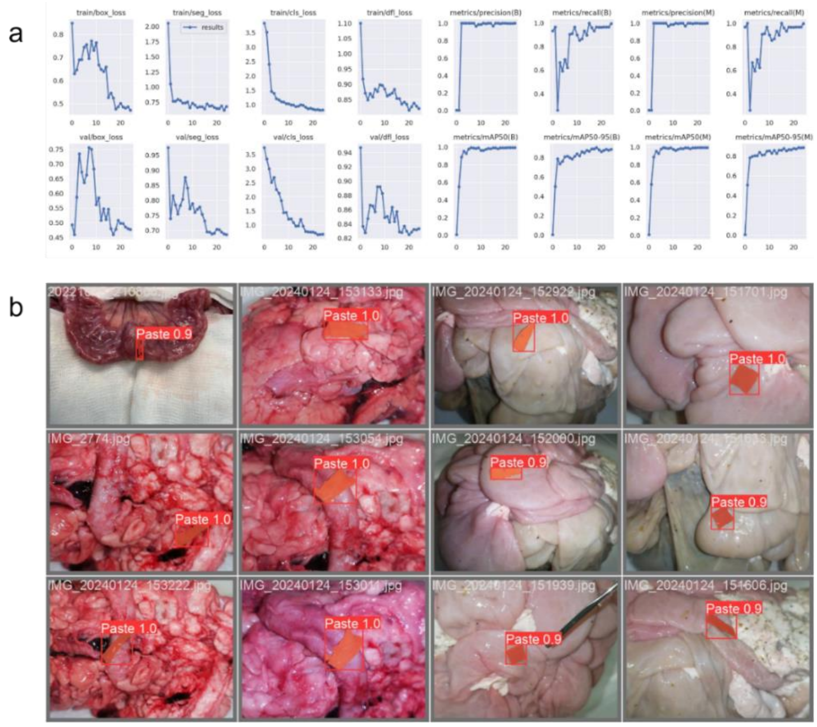

Figure S2: machine learning algorithm performance. *(a) Training and performance metrics of the paste detection and segmentation algorithm. (b) Images from the validation set with the paste being segmented correctly.*

In vivo minimally invasive surgery with soldering

Additional images of the surgery being performed on the porcine model are shown in Figure S3. It can be noted how the paste can be placed on the desired location (Figure S3a), how the soldering starts when the paste is recognized (Figure S3b), and how the fibers can be moved with graspers to reach areas that are difficult to reach (Figure S3c), such as the lateral abdominal wall (Figure S3d).

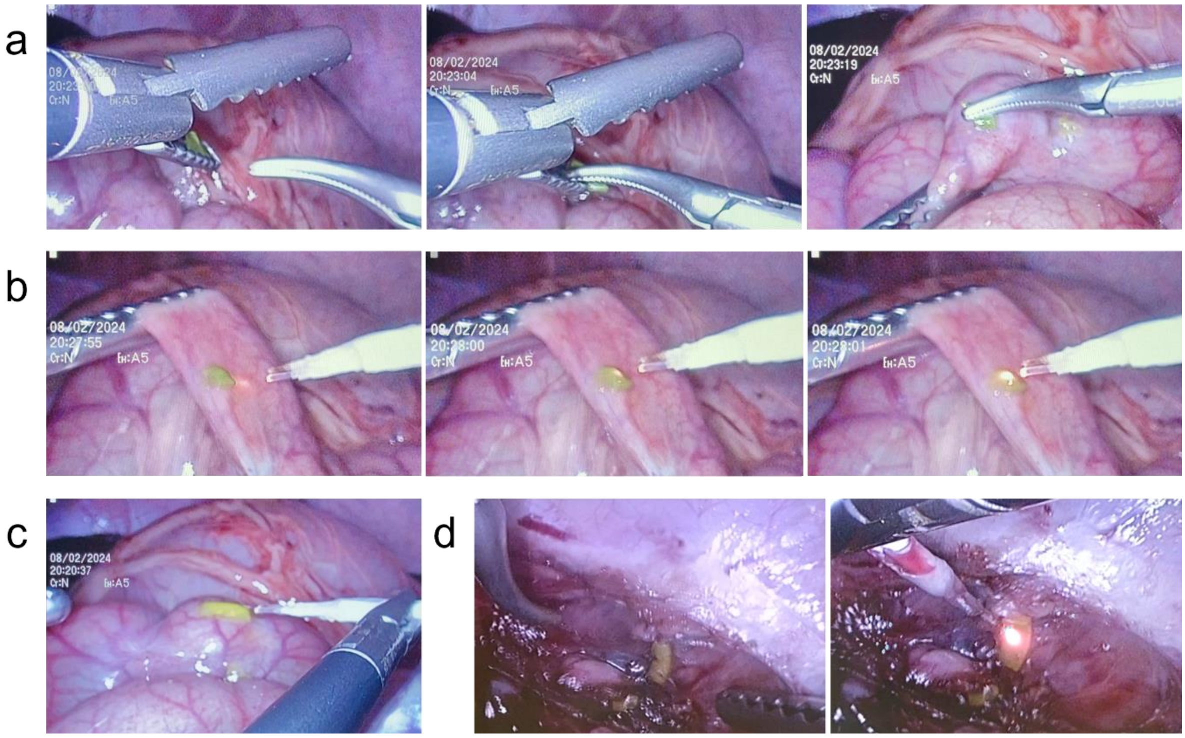

***Figure S3: in vivo laparoscopy images of soldering in chronological order**. (a) Positioning of the paste using the graspers. (b) Soldering of the paste on pig intestine, which starts only after paste detection, as seen from the laser intensity increase on the right image. (c) Fiber positioning using a grasper. (d) Positioning and soldering of paste on the lateral abdominal wall.*

Tensile strength measurements

The primary goal of laser tissue soldering is to replace sutures in the repair of soft tissues; therefore, its properties should primarily be compared with those of traditional suturing methods. Nevertheless, the tensile strength of intact porcine liver strips is included here as a control for comprehensive comparison (Figure S4). Additionally, the stress-strain curves for each sample category are provided in Figure S4b. In these curves, it is evident that the sutured samples exhibit dips in stress before reaching their ultimate tensile strength, a result of localized tearing in the tissue caused by the suture wire. In contrast, samples treated with solder paste maintain tissue integrity up to the point of adhesive failure. This is reflected in a single dip following the ultimate tensile strength, without prior dips, indicating a cohesive failure of

the soldered bond rather than tissue tearing. The stress-strain data were obtained using a Zwick Z010 uniaxial testing machine equipped with a 20 N load cell and operated at a speed of 20 mm/min to ensure controlled and consistent loading.

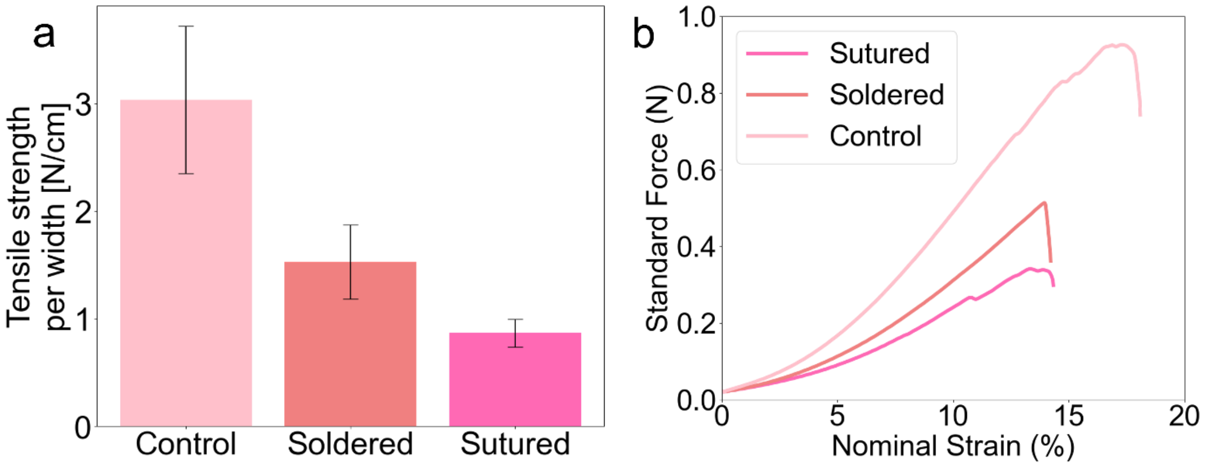

Figure S4: supplementary tensile strength data. *(a) Tensile strength measurements, including data from intact liver strips used as controls, illustrate the relative strength of each sample type. (b) Representative stress-strain curves for each sample category, providing a comparison of mechanical properties and deformation characteristics under applied stress.*

Histological data

Additional histological data (Figure S5) show the bonding efficacy of the solder paste on intestinal tissue. The images reveal strong adhesion with no visible tissue damage or detachment, demonstrating a stable and uniform bond that effectively closes the incision while preserving tissue integrity.

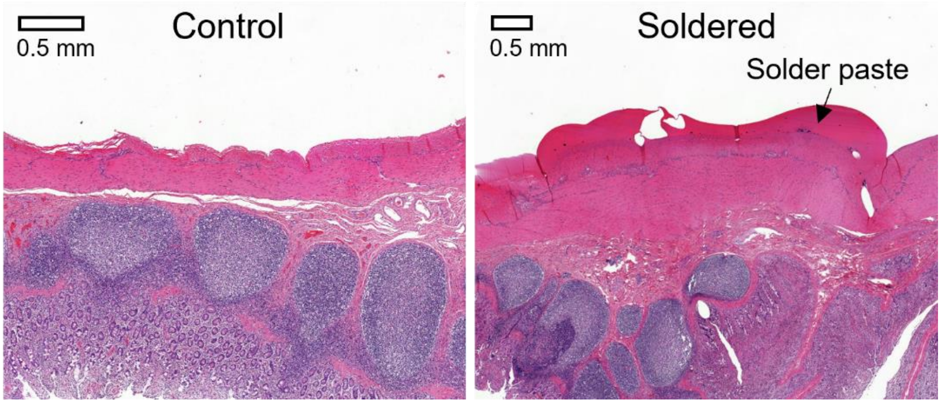

Figure S5: histology of soldered intestine. *Histological analysis of a porcine intestine is shown. Good adhesion and no visible thermal damage are present.*

Adhesion on dry tissues

Adhesion on dry tissues is outside the primary scope of this study, as it does not directly relate to our intended application. However, for completeness, we investigated how surface wetness impacts soldering adhesion. In this context, tensile strength on skin, which is drier than liver tissue, is compared. As shown in Figure S6, drier environments yield slightly stronger bonds, indicating that surface moisture plays a role in adhesion strength. In contrast to the liver measurements, porcine skin strips with a width of 0.5 cm were used in this experiment, with all other experimental conditions kept the same.

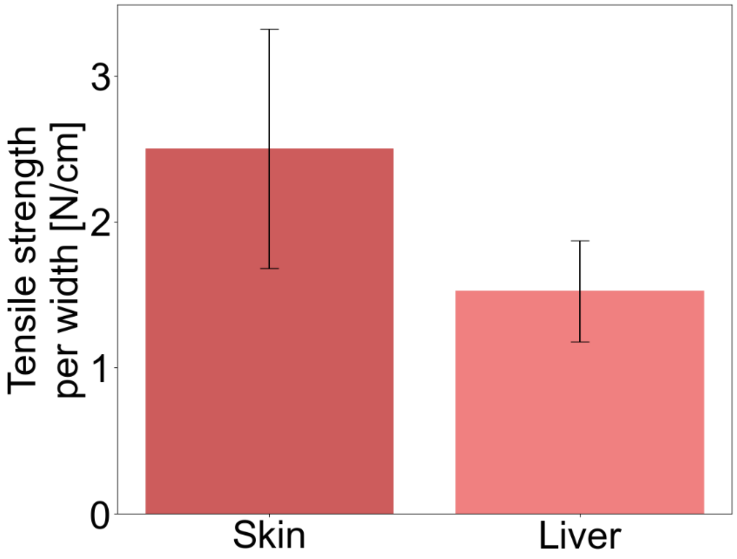

Figure S6: Impact of surface wetness on soldering adhesion. *Tensile strength measurements on skin and liver tissues illustrate how surface moisture affects bond strength. Skin, being drier than liver, achieves slightly stronger adhesion.*
